# Genome Sequence of Uric Acid-Fermenting *Eubacterium angustum* DSM 1989^T^ (MK-1)

**DOI:** 10.1128/genomeA.01439-16

**Published:** 2017-01-12

**Authors:** Anja Poehlein, Michael Y. Galperin, Jan R. Andreesen, Rolf Daniel

**Affiliations:** a Genomic and Applied Microbiology & Göttingen Genomics Laboratory, Institute of Microbiology and Genetics, Georg-August University, Göttingen, Germany; b National Center for Biotechnology Information, National Library of Medicine, National Institutes of Health, Bethesda, Maryland, USA; c Institute of Biology/Microbiology, University of Halle, Halle, Germany

## Abstract

*Eubacterium angustum* DSM 1989^T^ (MK-1) is a strictly anaerobic and uric acid-, xanthine-, and guanine-fermenting organism isolated from sewage sludge. The draft genome consists of one circular chromosome (2.4 Mb) and harbors 2,397 predicted protein-encoding genes.

## GENOME ANNOUNCEMENT

Only a few organisms are able to use purines as sole sources of carbon, nitrogen, and energy (1). Among these, anaerobic spore-forming bacteria *Gottschalkia acidurici* (formerly *Clostridium acidurici*) (2–4), *Clostridium cylindrosporum* (2, 3, 5), and *Gottschalkia purinilytica* (formerly *Clostridium purinilyticum*) (6) are obligately purinolytic, i.e., are nutritionally restricted to the degradation of purines (7). *Eubacterium angustum* is similar to these organisms in its nutritional properties but is unable to form spores, and it therefore has been classified into a different genus (8). *E. angustum* is a strictly anaerobic Gram-positive, non-spore-forming, and nonmotile bacterium, although flagella have been described (8). It is able to ferment uric acid, xanthine, and guanine. *E. angustum* has been isolated from sludge of the sewage plant in Göttingen, Germany (8). Here, we report the genome sequence of the type strain *E. angustum* DSM 1989 (MK-1).

Chromosomal DNA of *E. angustum* DSM 1989^T^ (MK-1) was isolated using the MasterPure complete DNA purification kit (Epicentre, Madison, WI, USA). The extracted DNA was used to generate Illumina shotgun paired-end sequencing libraries, which were sequenced with a MiSeq instrument and the MiSeq reagent kit version 3, as recommended by the manufacturer (Illumina, San Diego, CA, USA). Quality filtering using Trimmomatic version 0.32 (9) resulted in 2,751,284 paired-end reads. The assembly was performed with the SPAdes genome assembler software version 3.9.0 (10). The assembly resulted in 93 contigs (>500 bp) and an average coverage of 225-fold. The assembly was validated and the read coverage determined with QualiMap version 2.1 (11). The draft genome of *E. angustum* DSM 1989^T^ (MK-1) consists of a single chromosome (2,404,661 bp), with an overall G+C content of 43.67%. Automatic gene prediction and identification of rRNA and tRNA genes were performed using the software tool Prokka (12). The draft genome contained 9 rRNA genes, 73 tRNA genes, 1,764 protein-encoding genes with predicted functions, and 633 genes coding for hypothetical proteins.

In contrast to *Gottschalkia acidurici* 9a (13), the genome of *E. angustum* DSM 1989^T^ harbors a gene cluster encoding the glycine reductase complex, which can be used for energy conservation. The gene arrangement in the cluster is very similar to that in other purinolytic bacteria, such as *C. cylindrosporum* DSM 605 (14) and *G. purinilytica* DSM 1384 (15), and in the amino acid-degrading *Eubacterium acidaminophilum* DSM 3953 (16) and* Clostridium litorale* DSM 5388 (17). Analysis of the genome also revealed the genes encoding electron-bifurcating formate dehydrogenase, which has been recently described in *G. acidurici* (18). This cluster shows an organization very similar to that found in *G. acidurici* (13) and *G. purinilytica* (15) but lacks the second copy of the formate dehydrogenase gene (*fdhF*). The cluster from *E. angustum* DSM 1989^T^ also does not contain the second copy of the rubredoxin gene (*rbr*), which is present in the genome of *G. purinilytica* (15). The genome sequence of *E. angustum* will be used to clarify the phylogenetic position of this organism.

### Accession number(s).

The whole-genome shotgun project has been deposited at DDBJ/ENA/GenBank under the accession no. MKIE00000000. The version described here is version MKIE01000000.
